# Preparation of Mesoporous Analcime/Sodalite Composite from Natural Jordanian Kaolin

**DOI:** 10.3390/ma17194698

**Published:** 2024-09-25

**Authors:** Muayad Esaifan, Fayiz Al Daboubi, Mohammed Khair Hourani

**Affiliations:** 1 Department of Chemistry, Faculty of Art and Sciences, University of Petra, Amman 11196, Jordan; 2 Department of Chemistry, The University of Jordan, Amman 11942, Jordan; fayezaldaboubi112@gmail.com (F.A.D.); mhourani@ju.edu.jo (M.K.H.)

**Keywords:** meso-macroporous, zeolites, composite, analcime, sodalite, soft template, hydrothermal

## Abstract

In this work, a meso-macroporous analcime/sodalite zeolite composite was produced by a hybrid synthesis process between a complex template method and hydrothermal treatment at 220 °C of naturally abundant kaolinitic-rich clay, using dodecyltrimethylammonium bromide as an organic soft template to enhance the mesoporous structure. The chemical and morphological properties of the developed zeolites composite were characterized using powder X-ray diffraction (PXRD), attenuated total Reflectance–Fourier transform infrared spectroscopy (ATR-FTIR), thermogravimetric analysis (TGA), N_2_ adsorption/desorption; and scanning electron microscopy with energy dispersive spectroscopy (SEM-EDS) methods were used to study the morphology, chemical composition and structure of the product. Two types of zeolite particles were obtained:(1) hollow microsphere with an attached analcime icositetrahedron of 30–40 µm in size and (2) sodalite microsphere with a ball-like morphology of 3–4 µm in size. Both N_2_ adsorption/desorption and surface area data confirmed the high potentiality of the produced zeolite composite to act as an excellent adsorbent to remove inorganic pollutants such as Cu, Cd, Cr, Ni, Zn, and Pb ions, organic pollutants such as dyes, phenolic compounds, and surfactants from water; and their high catalytic activity, especially in the oxidation reaction of volatile organic compounds. The catalytic activity and adsorption ability of the produced analcime/sodalite composite will be tested experimentally in future work.

## 1. Introduction

Developing new mesoporous materials and exploring their use in various fields such as areas of catalysis [1,2], biomedical [3,4,5], adsorption [6,7], gas sensing [8,9], drug delivery [10,11,12,13], and water purification [14,15,16] is of prime importance due to their unique properties, such as large surface area, tunable pore size, and high pore volume [17]. According to the IUPAC, mesoporous materials are defined as materials with apore size in the range of 2−50 nm [18]. The first highly ordered mesoporous silicate designated as MCM-41 was reported in 1992 and synthesized using the concept of “templating” by hydrothermal reaction of aluminosilicate gels in the presence of quaternary ammonium cationic surfactants [19]. Extensive studies have been conducted to develop new classes of porous materials, combining the unique features of both microporous zeolites and macroporous materials that exhibit a unique ordered mesoporous structure, a high surface area (600–1000 m^2^∙g^−1^), a large pore diameter (4–30 nm), and a high pore volume (0.5–2 cm^2^∙g^−1^), such MCM-48 and SBA-15 [20,21].

The key factor in fabricating mesoporous materials with interconnected channels is selecting the suitable synthetic protocols. These methods are known as soft, hard, and complex templates. In the soft templates methodology, additives such as organic-based supramolecules are involved in the synthesis process. Subsequently, these are removed by thermal treatment. The hard templates methodology involves leaching additives such as zeolites and silica by acid or basic treatment. Complex templates are a combination of soft and hard templates, two hard templates, or two soft templates of different length scales. The complex templates have been successfully used to prepare hierarchically bi-modal and tri-modal meso-macroporous materials with interconnected pore channels [22].

Among the inorganic mesoporous materials that have been researched, synthetic mesoporous zeolites are considered among the most eco-efficient mesoporous materials. This is attributed to the unique properties of zeolites, like their high ion exchange capacity, affinity for heavy metal cations, chemical stability, and mechanical sturdity [14,23,24,25,26].Several studies reported the successful synthesis of mesoporous zeolites using low-cost manufacturing methods and starting from naturally abundant materials, including kaolinite-rich clay [14,27,28,29] and basalt powder [30]. This method is based on using these naturally abundant materials as a source of solid precursors that can release reactive species of silicon and alumina that can react with alkaline activating solutions such as sodium hydroxide or potassium hydroxide to produce different types of zeolites. Either natural or synthetic zeolites. Dran’kov et al. [31] also reported the synthesis of nanostructured magnetite (Fe_3_O_4_) and supported zeolite as an adsorbent material composed of magnetic Fe_3_O_4_-zeolite framework with a high removal efficiency of cesium and strontium ion from contaminated water. The use of natural zeolites to immobilize radioactive cesium ions was reported by Shichalin et al. [32] via spark plasma sintering of the loaded cesium ion on zeolite. In all previous studies, the catalytic activity of zeolite materials has been attributed to the acidic sites on the inner pore surface [33].

The main aim of the present work is to develop a facile and eco-efficient method for synthesizing a novel, highly crystalline and ordered mesoporous analcime/sodalite zeolite composite using dodecyltrimethylammonium bromide as an organic surfactant template. Although there are many works in the literature on the synthesis of zeolite analcime with different starting materials and different methods [34,35,36,37], to the best of our knowledge, in this work, the production of analcime/sodalite zeolites composite using a hybrid synthesis process between a complex template method and hydrothermal treatment of naturally abundant aluminosilicate material (kaolinite) is reported for the first time. The physicochemical and morphological properties of the resultant material were also systemically investigated by various techniques, and the hierarchical pores’ structure, in addition to the surface area, was discussed as potential adsorbent materials for various environmental remediation.

## 2. Materials and Methods

**Figure 1:**
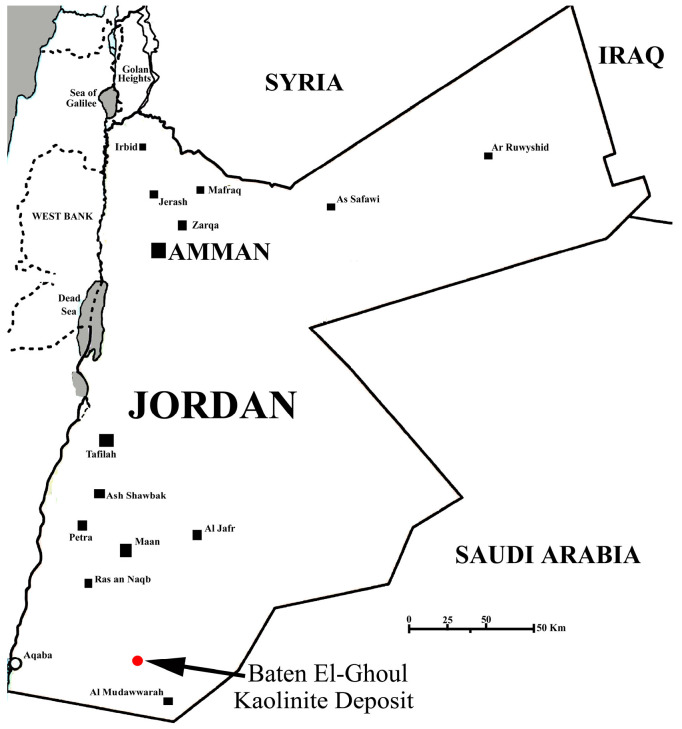
Geological map of the Baten El-Ghoul kaolinite deposit and sampling points.

A sample of natural kaolinitic clay was collected from the Baten El-Ghoul deposit, which is located in the southern part of Jordan (Figure 1), and used as a solid precursor for the synthesis of analcime/sodalite composite material. The mineralogical, chemical, and morphological properties of this clay sample were examined and reported elsewhere [38]. According to Esaifan et al. [38], the major component (~68%) in this sample was kaolinite, Al_2_O_3_∙2SiO_2_∙2H_2_O. The other components include quartz (SiO_2_), calcite (CaCO_3_), hematite (Fe_2_O_3_) and illite (K,H_3_O)(Al,Mg,Fe)_2_(Si,Al)_4_O_10_[(OH)_2_,(H_2_O)]). Dodecyl trimethylammonium bromide (99%—ACROS Organics, Waltham, MA, USA) was used as the organic template for pore enlargement to obtain a reticular mesoporous composite structure. Sodium hydroxide (NaOH, 99.0%, Loba Chemie, Mumbai, India), ethanol (Gainlland chemical company, Deeside, UK). All solutions were made from the above-mentioned reagents without further purification. The reagents were dissolved in Milli-Q water (Millipore Advantage 10, Millipore, Merck KGaA, Darmstadt, Germany). All experiments were performed at ambient conditions unless mentioned otherwise.

The mineralogical components and structural phases changes were investigated using a Shimadzu MAXima X XRD-7000 X-ray diffractometer (XRD) with a Cu Ka-radiation k = 1.54 Å, 40 kV, 40 mA at a 2h range 2–70 with a scan rate of 2 deg/min, step scan size 0.02, and a receiving slit of 0.3 mm (Kyoto, Japan). The morphology and chemical composition of the raw kaolin starting material and the synthesized product were investigated using a Phenom XL G2 scanning electron microscope combined with an AXS EDS system (Thermo Fisher Scientific, Waltham, MA, USA). The kaolinitic clay and the synthesized product were coated with a ~300 Å thick platinum film under an argon atmosphere using an AGAR sputter coater machine (model AGB7340, Essex, UK) in a high-vacuum evaporator. The SEM images were produced at 6 × 10^−4^ Pa and 15 kV accelerating voltage.

An FTIR spectrometer (Lambda Scientific, Adelaide, Australia) and an attenuated total reflectance diamond crystal unit were used to obtain the infrared spectra. The spectra were recorded by scanning from 4000 to 650 cm^−1^ with a resolution of 4 cm^−1^. The surface area and pore size distribution of the synthesized zeolite composite were investigated using N_2_ adsorption/desorption isotherm measurements. An N_2_ adsorption-desorption analyzer (Quantachrome Instruments, Anton Paar—Autosorb-iQ model, Miami, FL, USA). The samples were exposed to a degassing process under a vacuumed environment for three hours at 250 °C. Adsorption/desorption data were processed using the multi-point Brunauer–Emmett–Teller method (BET). The surface area and pore size distribution data were collected using the AsiQwin software, version 5.21, and the non-local density functional theory model (NLDFT). The standard slit-pore model for N_2_ adsorption at 77 K on silica was selected because it is widely used to characterize crystalline materials.

The thermogravimetric analysis (TGA) experiments were carried out using a NETZSCH STA 409 PG/PC Instrument (Selb, Germany) under a stream of nitrogen gas (G5, 99.999% minimum purity, International Company for Industrial and Medical Gases, Jordan). The samples were placed in alumina crucibles and heated from ambient temperature to 900 °C at a heating rate of 20 °C/min under a flow rate of 30 mL/min of nitrogen stream.

The synthesis of mesoporous analcime/sodalite composite was carried out by adding 150 mL of Milli-Q water to 50.0 g of kaolin and 25.0 g of NaOH pellets. The mixture was placed in a round-bottom flask equipped with a reflux. The mixture was refluxed at ~100 °C and stirred at 1000 rpm for one hour. The heat was uniformly distributed by setting the round-bottom flask in a sand bath over a hotplate. A total of 0.5 g of dodecyl trimethylammonium bromide surfactant was dissolved in 50.0 mL ethanol. The prepared surfactant solution was added to the refluxed mixture. The final mixture was refluxed for another 1.0 h with continuous stirring at 1000 rpm. The solution was subjected to suction filtration. The filtrate was hydrothermally autoclaved in a P.T.F.E lined, stainless steel hydrothermal synthesis reactor at 220 °C for three hours. The precipitate was collected and extensively rinsed with Milli-Q water. After washing the precipitate, it was dried in an oven at 105 °C for one day. The organic surfactant was removed by the calcination of the resulting product at 500 °C for three hours using a muffle furnace (Nabertherm model L 24/11 BO, Lilienthal, Germany).

## 3. Results

### 3.1. Chemical and Mineralogical Characterization of the Synthesized Zeolites Composite

**Figure 2:**
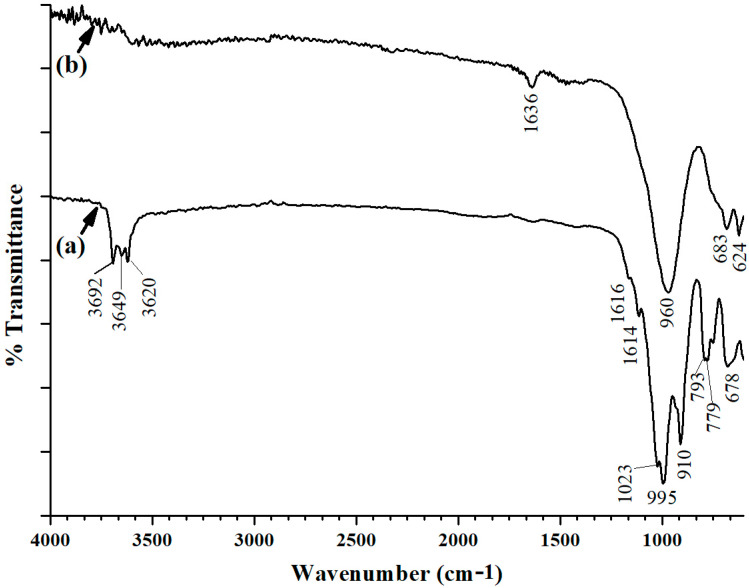
Infrared spectra of the (**a**) kaolin starting material and (**b**) synthesized zeolites composite.

The chemical structure of the developed materials was determined and confirmed using infrared spectroscopy and powder X-ray diffraction. The infrared spectra of both the raw material and synthesized material indicate a drastic change in IR spectra, as depicted in Figure 1. A comparison of Figure 2b for the prepared zeolites composite with that of the starting material (Figure 2a) indicates that there is a real transformation to a new material.

The peak observed at 3620–3692 cm^−1^ range in the infrared (IR) spectrum for the kaolinitic raw material corresponds to the stretching vibrations of the hydroxyl group (-OH). This band is not observed for the prepared zeolites composite. Three bands that are observed between 600–1650 cm^−1^ merged into a single IR band centered at 960 cm^−1^. These peaks are attributed to Si-O-Si and Si-O-Al bonds, which merge to form a more homogeneous structure in the spectrum of the formed material. This indicates that the silicate and aluminate species merge into a more uniform silicon-aluminum structure similar to that found in zeolites.

**Figure 3:**
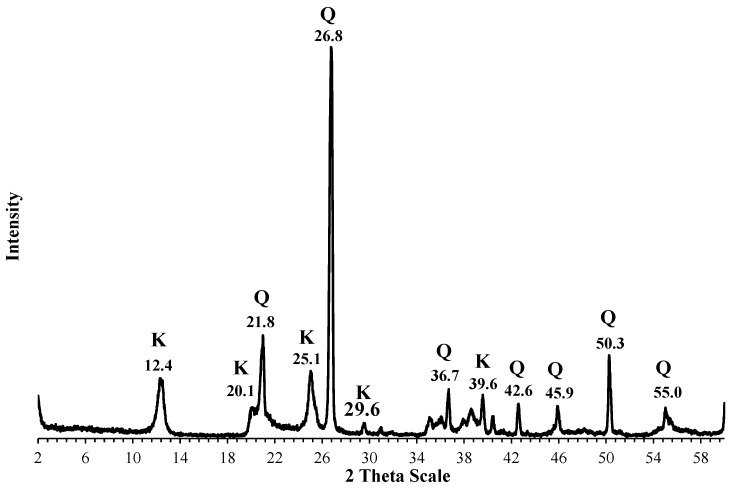
XRD diffractogram of thekaolinitic-rich clay (K: kaolinite, Q: quartz).

**Figure 4:**
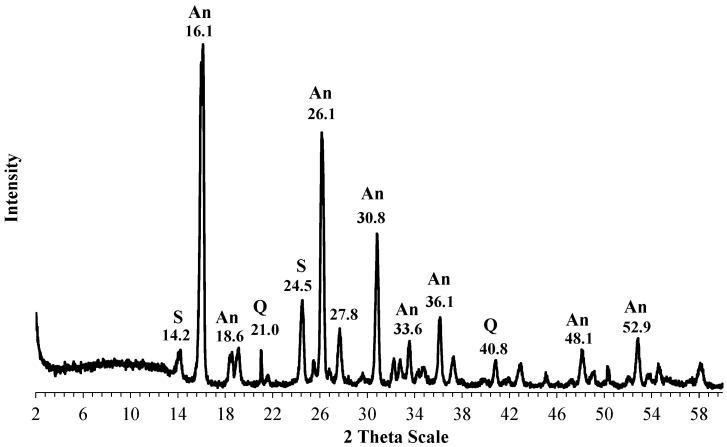
XRD diffractogram of the synthesized zeolites composite (S: sodalite, An: analcime, Q: quartz).

Thepowder XRD diffractograms for the kaolin starting material and the synthesized zeolites composite are displayed in Figure 3 and Figure 4, respectively. Figure 3 shows a typical diffractogram for a kaolinite-rich clay, i.e., a material that contains a large fraction of kalinite, quartz, and a fraction of noncrystalline materials. Figure 4, on the other hand, shows well-defined peaks indicating the existence of new crystalline substances. Close inspection of the peaks in Figure 4 indicates the presence of analcime (a zeolite), while the other peaks are attributed to the presence of sodalite, in addition to the complete disappearance of the peaks related to kaolinite. Thus, the XRD patterns suggest that the hydrothermal process led to the transformation of the kaolinitic clay into analcime and sodalite composite. This conclusion is also supported by the JCPDF databases.

**Figure 5:**
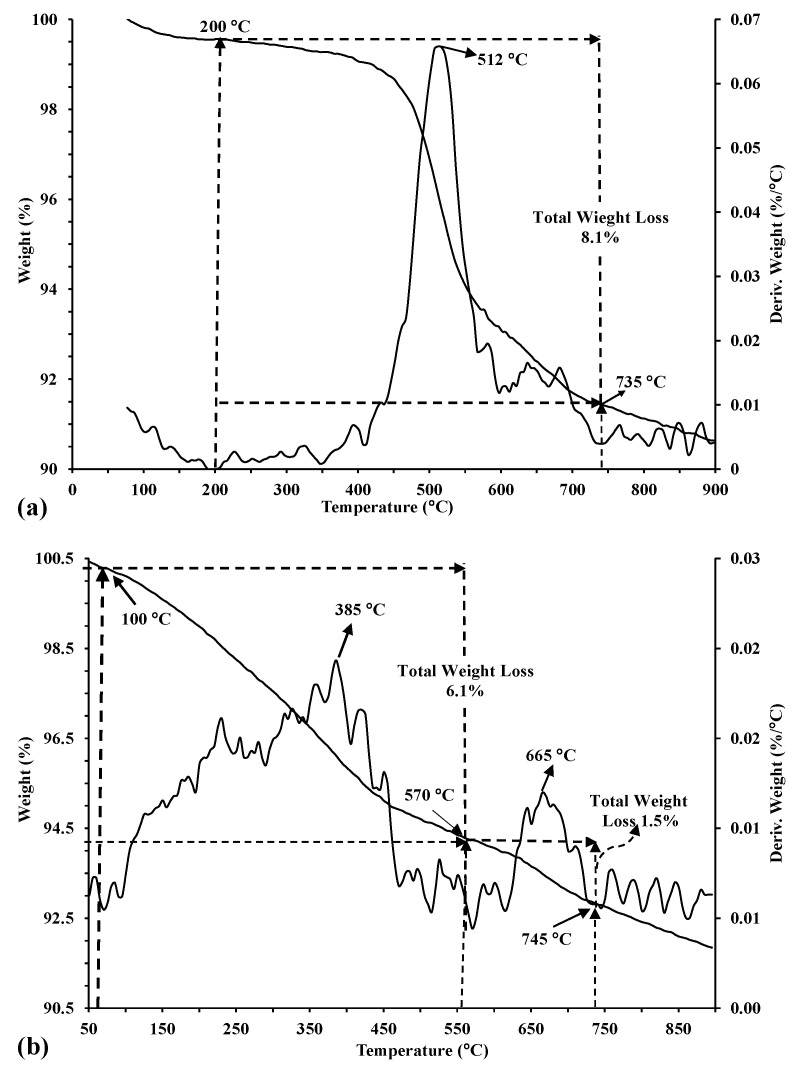
TGA and DTG curves of the (**a**) kaolinitic-rich clay and (**b**) synthesized zeolites composite.

The thermal stability of the kaolinitic-rich clay and synthesized zeolites composite were investigated using TGA and DTG analyses;their curves are illustrated in Figure 5a and Figure 5b, respectively. One sharp endothermic peak was observed for the kaolinitic-rich clay (Figure 5a) at heating temperatures ranging between 200–735 °C to achieve a maximum peak at 512 °C with a total weight loss of 8.1%. According to Esaifan et al. [38], this endothermic step is attributed to the dehydroxylation of kaolinite and corresponds to a kaolinite content of 68%. Two-step weight loss was observed on the TGA curve of the synthesized zeolite composite (Figure 5b). For the synthesized zeolite composite (Figure 5b), the TGA curve showstwo-step weight losses, indicating the presence of two zeolite types. Obvious weight losses were attributed to molecular structural water (known as zeolitic water), with values of 6.1% and 1.5% for analcime and sodalite, respectively. A total dehydration of analcime occurred at about 385 °C, which was comparable to the natural analcime [38], but that of sodalite was as high as 665 °C, indicating better thermal stability.

### 3.2. Morphological Characterization of the Synthesized Zeolites Composite

**Figure 6:**
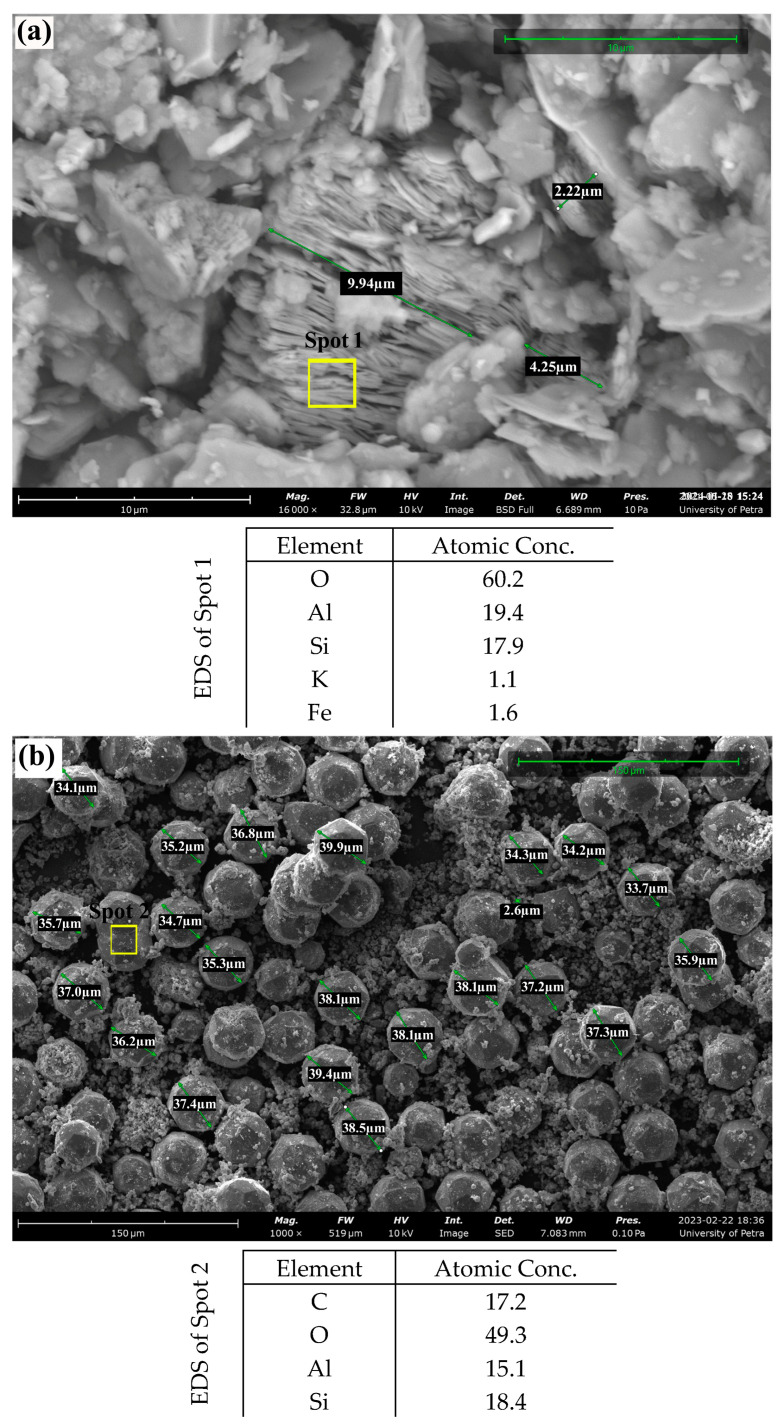

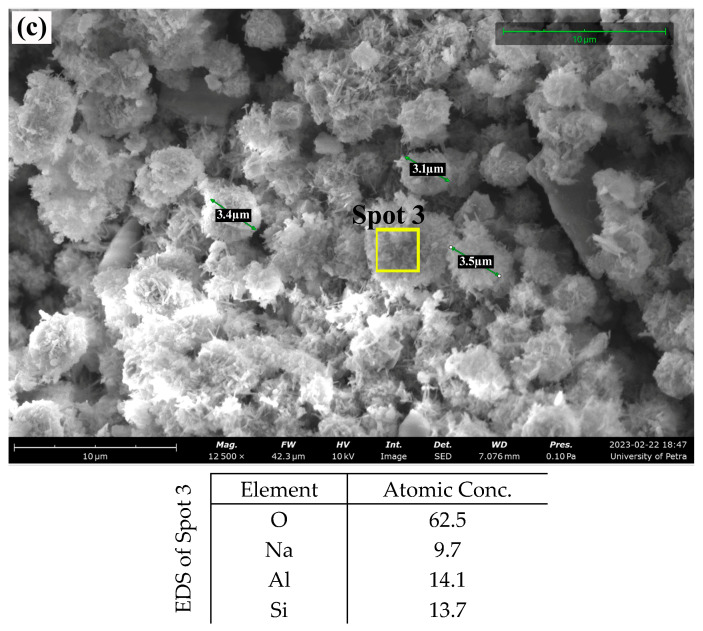
SEM micrographs of (**a**) kaolinitic-rich clay, (**b**) synthesized zeolites composite with 1000× magnification, and (**c**) synthesized zeolites composite with 12,500× magnification.

Figure 6 shows the SEM micrographs and the results for EDX spectral analysis for the kaolinitic clay starting material and the prepared zeolitic composite. The micrographs in Figure 6a show a flaky, nonhomogeneous, and layered kaolinitic clay, and EDS analysis displayed for kaolinitic clay starting material shows an Al-to-Si approximate ratio of 1.08, which is typical for natural kaolinite raw material. The micrographs for the synthesized analcime and sodalite composite show the complete disappearance of the former kaolinitic clay structures and the appearance of two distinct crystalline phases (Figure 6b,c). The first phase is the icositetrahedron analcime crystals with crystal size ranging between 30–40 µm (Figure 6b), and the EDS analysis for analcime displayed that the ratio of Si-to-Al is about 0.82, which is less than both the raw kaolinite and sodalite phase as adversely affected by the presence of carbon attributed to the residual surfactant in analcime crystal. Asit appears in Figure 6c, the second phase is composed of sodalite crystals with a wool ball-like morphology of 3–4 µm in size. The EDS analysis of this phase (Figure 6c) displayed a ratio of Si-to-Al equal to 1.03, which is higher than in the case of the analcime phase due to the inclusion of sodium ions in the sodalite structure instead of carbon, which is heavier.

### 3.3. Isotherm N_2_Gas Adsorption/Desorption Study of the Synthesized Zeolites Composite

**Figure 7:**
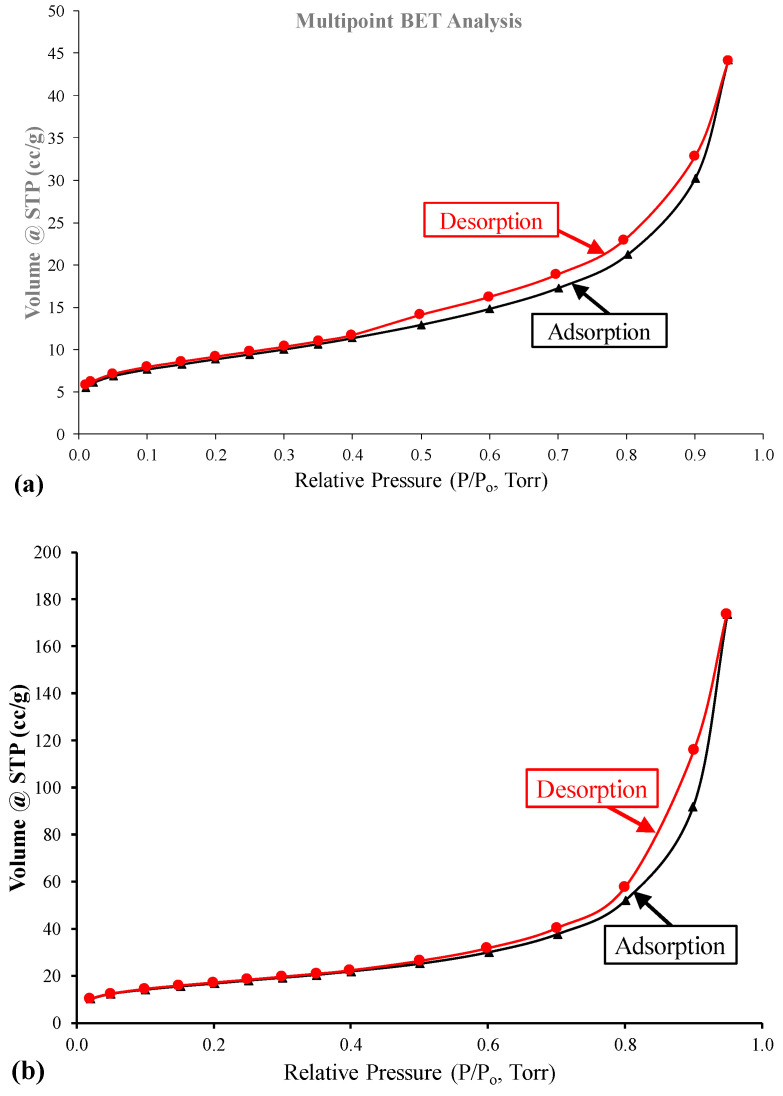
N_2_ adsorption-desorption isotherms for the (**a**) kaolinitic-rich clayand (**b**) synthesized zeolites composite.

The isotherm N_2_ gas adsorption/desorption study was conducted to confirm the high efficiency of the produced zeolite composite in acting as an adsorbent for inorganic and organic pollutants from water and their high catalytic activity. The isotherms N_2_ gas adsorption/desorption curves of the kaolinitic-rich clay starting material and the synthesized analcime/sodalite composite are displayed in Figure 7. A closer look at Figure 7a shows that the adsorption isotherm for the kaolinitic-rich clay starting material hasa great similarity between this isotherm and the IUPAC Class III isotherm [18]. This class is associated with physisorption by macroporous substances with weak interaction between the adsorbent and adsorbed molecules. The adsorbed molecules, in this case, tend to cluster around the best possible sites on the surface of the macroporous adsorbent. The N_2_ adsorption-desorption isotherm for the analcime/sodalite composite (Figure 7b) is of type IV, according to the IPUAC classification, with an H4 loop in the latter half part (P/P0 is 0.5~1.0), indicating that the product has a typical mesoporous structure. This type of loop is often found with zeolites of mesoporous and meso-macroporous materials [18].

**Figure 8:**
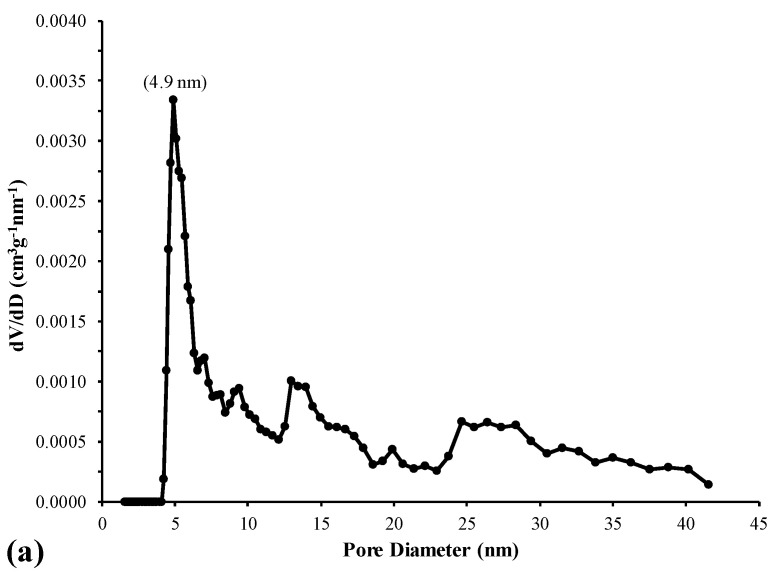

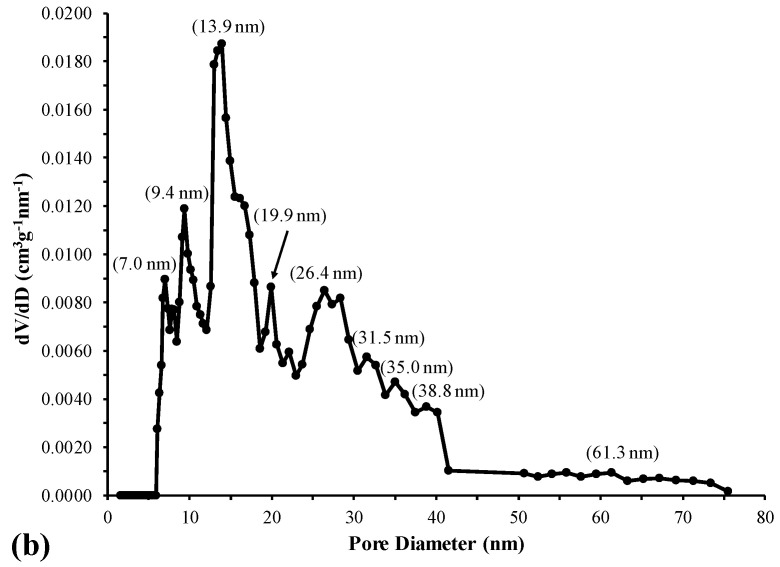
Pore size distribution curves, calculated using thedensity functional theory at STP for the (**a**) kaolinitic-rich clayand (**b**) synthesized zeolites composite.

The pore size distribution curves for the kaolinitic-rich clay starting material and the analcime/sodalite mixture are presented in Figure 8. The kaolinitic-rich clay is basically nano-porous with a mean size of 3.6 nm. The synthesized mesoporous analcime/sodalite mixture has a pore diameter between 4 and 75 nm with a mean value of 12.8 nm. This wide range of pore size might be due to the fact that the pore size in the zeolitic analcime is different from that in sodalite [39].

Table 1 shows the calculated specific surface areas for the kaolin starting material and the synthesized analcime/sodalite composite based on the Multi-BET, Langmuir, and NLDFT models. The calculated values of the specific surface areas for the kaolin samples range from 14.8 to 25.9 m^2^∙g^−1^ compared to 58.0 to 107.3 m^2^∙g^−1^ for the analcime/sodalite composite. These results are genuine manifestations of the transformation of the kaolinitic-rich clay to meso-macroporous zeolitic materials with adsorption capacities comparable to the most efficient adsorbents reported in the literature [14,23,24,25,26,27,28,29,40].

## 4. Conclusions

The present work was pivoted on the preparation of meso-macroporous zeolitic materials from kaolinitic-rich clays, which are abundant in Jordan. The main objective of this work was to prove the notion that Jordanian kaolin may be used as a source of silicon and aluminum for the preparation of zeolitic materials. The other objective was to use the surfactant dodecyl trimethylammonium bromide as a soft templating agent and pore expander. The preparation procedure followed the traditional hydrothermal procedure.

The produced composite was subjected to an extensive analysis to identify the resulting material(s), in addition to an analysis of the starting material for comparison. FTIR, SEM, EDX, BET, XRD, TGA, and BET were used to pursue this task. Allthese analytical tools showed that the starting material was transformed into an analcime/sodalite composite. The XRD analysis, for instance, revealed the appearance of peaks indicating the presence of analcime/sodalite composite and the disappearance of kaolinite distinctive peaks. The transformation of kaolinite into zeolitic phases was further confirmed by infrared spectra, where distinct vibration bands at 2500–650 cm^−1^ and 4000–2500 cm^−1^ in untreated clays vanished during the transition to the zeolite phase, leaving a distinctive band at 1000–1035 cm^−1^.

The SEM and EDX results indicated the presence of two types of crystals:the icositetrahedron crystals attributed to analcime and the wool-ball-like crystals attributed to sodalite. The EDX analysis revealed Si-to-Al percentages for analcime/sodalite composite that are different from the kaolinitic starting material and close to the values reported in the literature. The N_2_ adsorption/desorption isotherms of the synthesized analcime/sodalite composite indicated a meso-macroporous structure. The raw kaolin starting material exhibited surface area values (BET, DR, and BJH) ranging from 4 to 20 m^2^/g, while the prepared meso-macroporous zeolitic composite showed values between 23 and 37 m^2^/g. Both the adsorption capacity and surface area data confirmed the ability of the produced zeolite composite to act as an excellent adsorbent to remove both inorganic and organic pollutants from water and their high catalytic activity. The successful use of naturally abundant kaolin to synthesize zeolite composites in comparison to the use of commercial pure chemicals as starting materials can significantly reduce the production costs of such adsorbents, with an estimated price of the produced zeolite composite ranging from 2–5 US$ per kg.

## Figures and Tables

**Table 1:** Surface area values of thekaolinitic-rich clay and synthesized zeolites composite.

Sample	BET ^a^ (m^2^·g^−1^)	SBET ^b^ (m^2^·g^−1^)	Langmuir ^c^ (m^2^·g^−1^)	NLDFT ^d^ (m^2^·g^−1^)	V_micro_^e^ cm^3^/g	V_total_^f^ cm^3^/g
Kaolinitic-rich clay	14.9	25.3	15.9	25.9	0.0015	0.068
Zeolites composite	58.0	56.9	107.3	61.5	0.0345	0.269

^a^ Surface area, calculated using the multilayer BET model. ^b^ Surface area, calculated using the single point BET model. ^c^ Surface area, calculated by the Langmuir model. ^d^ Surface area, calculated using the non-linear density functional theory. ^e^ Micropore volume, calculated using t-method. ^f^ Total pore volume at P/P° = 0.95.

## Data Availability

The raw data supporting the conclusions of this article will be made available by the authors on request.
